# Clinical Remarks. Professor Thomas F. Rochester, on Œdema Glottidis

**Published:** 1871-01

**Authors:** F. Bradnack

**Affiliations:** Member of the Class


ART. VI.—*Clinical Remarks.* Professor THOMAS F. ROCHESTER *on Œdema Glottidis.*

REPORTED BY F. BRADNACK, MEMBER OF THE CLASS.

The case just observed in the ward is a case of œdema glottidis. In this affection the dyspnœa which exists is generally referred to the epigastric region, and this remark likewise applies to most glotteal diseases. Œdema glottidis is seldom a primary disease. Persons subjected to the inhalation of steam, generally die from this affection. It frequently complicates variola. In like manner it is occassionally a concomitant of measles. In the case just now under consideration, there was a sort of diphtheritic appearance on the tongue. Now, what is to be done in this affection? Manifestly, we must endeavor to remedy it as speedily as possible, the condition being one of great gravity; and if there be much delay in affording relief, patients will frequently die from carbonization of the blood, the passage to the lungs being oppilated, or perhaps entirely occluded. Topical treatment consists in the scarification of the glottis, by a probe-pointed bistoury; but it is difficult to make the cuts deep enough to remedy the œdema without simultaneously doing injury. Dr. Buck, of New York, has invented an instrument for performing this operation without danger. Patients should not be allowed to die from œdema. To prevent such a result, either laryngotomy or tracheotomy may be performed. Of these operations the former is the easier and the better. A probe may be left in the wound, or a tracheal tube may be inserted, according to circumstances. It is, of course, of great importance to ascertain whether the obstruction is in the larynx or in the trachea. In the case referred to, three leeches were applied to the sides of the trachea. If leeches do not relieve in an hour, the practitioner should stand prepared to operate. In this connection it may be remarked that tracheotomy is by no means so trivial an operation as is generally imagined. When there is great pain with œdema glottidis, opium may be given with a double object: first, for its anodyne, and, secondly, for its antiphlogistic effects. For these purposes, full doses should be given. Where there is a tendency to what are known as head symptoms, the operation of the opium should be watched closely, lest stupor or dullness supervene. But in this disease opium often relieves the œdema, and the patient gets the benefit of its antiphlogistic, without its narcotic effects. In these cases prompt and decisive action is of the utmost importance. Complicating œdema glottidis may be spasm of the glottis. Opium controls greatly this tendency to spasm. You may ask why mercury is not used where thickening of the glottis would appear to make its use advisable. But here there is not time to produce the effects of mercury.

The following incident may serve to illustrate the necessity of prompt and energetic action in this affection:—Several years ago, when Professor Frank H. Hamilton had charge of the surgical department of this hospital, as I was one day passing through one of the surgical wards, a patient, lying in bed, beckoned to me. On asking the *interne* what was the matter with the man, the latter replied that there was nothing particularly the matter with him. But the man's look was so imploring, and his gestures so beseeching, that I went to his bedside. He spoke with a labial tone, being scarcely able to articulate at all. He said that he was choking to death. His voice was husky, and he appeared then to be laboring under laryngitis, rather than œdema glottidis. At twelve o'clock that night I was called in great haste to the hospital. On entering the door, and hearing the breathing of the patient, I at once realized his condition. I learned by brief inquiries that at one o'clock P. M. he began to suffer from great dyspnœa, and so violent were his efforts to obtain relief that in one of his paroxysms he had jumped out of bed to the floor, and there fallen in a state of syncope, which syncope, by the way, probably saved his life, by relaxing temporarily the muscular system. There being no proper surgical instruments then at hand, and the case being altogether too urgent to allow of sending for any, with a dull knife I immediately made an incision, about an inch in length, into the larynx, and pulled open the orifice with probes, and finally inserted a tracheal tube. The relief was instantaneous. The man wore the tube six weeks, and other treatment being applied, the case eventuating in perfect recovery.

